# CINCINNATA-Like TCP Transcription Factors in Cell Growth – An Expanding Portfolio

**DOI:** 10.3389/fpls.2022.825341

**Published:** 2022-02-22

**Authors:** Monalisha Rath, Krishna Reddy Challa, Kavitha Sarvepalli, Utpal Nath

**Affiliations:** ^1^Department of Microbiology and Cell Biology, Indian Institute of Science, Bengaluru, India; ^2^Undergraduate Program, Indian Institute of Science, Bengaluru, India

**Keywords:** cell expansion and differentiation, cell proliferation, CIN-TCP, Arabidopsis, hypocotyl, cotyledon, leaf

## Abstract

Post-mitotic cell growth is a key process in plant growth and development. Cell expansion drives major growth during morphogenesis and is influenced by both endogenous factors and environmental stimuli. Though both isotropic and anisotropic cell growth can contribute to organ size and shape at different degrees, anisotropic cell growth is more likely to contribute to shape change. While much is known about the mechanisms that increase cellular turgor and cell-wall biomass during expansion, the genetic factors that regulate these processes are less studied. In the past quarter of a century, the role of the CINCINNATA-like TCP (CIN-TCP) transcription factors has been well documented in regulating diverse aspects of plant growth and development including flower asymmetry, plant architecture, leaf morphogenesis, and plant maturation. The molecular activity of the CIN-TCP proteins common to these biological processes has been identified as their ability to suppress cell proliferation. However, reports on their role regulating post-mitotic cell growth have been scanty, partly because of functional redundancy among them. In addition, it is difficult to tease out the effect of gene activity on cell division and expansion since these two processes are linked by compensation, a phenomenon where perturbation in proliferation is compensated by an opposite effect on cell growth to keep the final organ size relatively unaltered. Despite these technical limitations, recent genetic and growth kinematic studies have shown a distinct role of CIN-TCPs in promoting cellular growth in cotyledons and hypocotyls, the embryonic organs that grow solely by cell expansion. In this review, we highlight these recent advances in our understanding of how CIN-TCPs promote cell growth.

## Introduction

Plant growth and development are remarkably plastic, which helps them overcome the constraints posed by their sessile existence and enables them to thrive in diverse environmental conditions. Developmental plasticity is achieved partly by preserving the self-renewing stem cells within the meristem niches and by post-embryonic initiation of lateral organs from the flanks of the meristem (Powell and Lenhard, 2012). Organ growth is a resultant outcome of two key cellular processes, proliferation, and post-mitotic expansion, the former preceding the latter. Proliferation produces an adequate cell reserve at an early stage of organ growth, and expansion increases cell volume under the force of turgor pressure (Donnelly et al., 1999; Cosgrove, 2005; Gonzalez et al., 2012). For example, mitotic cycle in Arabidopsis and tobacco leaves ceases when their blades reach ∼10% of their final area, triggering endoreduplication-mediated cell expansion that drives the remainder of the blade growth (Poethig and Sussex, 1985; Donnelly et al., 1999).

Though the physical basis of cell expansion through turgor pressure and cell-wall extensibility has been studied in detail (Ray et al., 1972; Braidwood et al., 2014), its genetic regulation remains less clear. This is primarily because of an overlapping occurrence of cell division and expansion in the lateral organs of post-embryonic origin. For example, within the growing primordium of most angiosperm leaves, cell division and expansion coexist in the proximal and distal domains, respectively (Nath et al., 2003; Das Gupta and Nath, 2015). Cells transiting from an actively dividing state to differentiation characterize a dynamic transition zone, flanked by mitotic cells to its proximal end and differentiated cells to its distal end. Perturbations in rate, duration, or distribution of cell proliferation and expansion often affect the final size and shape of organs (Donnelly et al., 1999; Efroni et al., 2008; Andriankaja et al., 2012). Because proliferation and differentiation are linked by a compensatory mechanism (Gonzalez et al., 2012) in post-embryonic organs, it is difficult to uncouple the contribution of cell growth from that of cell division to the final organ morphology. However, the organs of embryonic origin such as hypocotyl and cotyledon serve as an ideal model for cell growth studies since cell division is absent or insignificant during their growth after germination (Tsukaya et al., 1994; Stoynova-Bakalova et al., 2004; Boron and Vissenberg, 2014). Besides, these embryonic organs possess simpler and uniform epidermal cell morphology, making their analysis more tractable. Utilization of these simplifying advantages of hypocotyl and cotyledon has shed light on the molecular players involved in unidirectional and planar cell expansion, respectively. In this review, we highlight recent advances in our understanding of the genetic regulation of post-mitotic cell growth, with an emphasis on CIN-TCP transcription factors and their new-found role in cell expansion.

## Types of Cell Growth

Because the rigid wall precludes migration of plant cells, organ morphology is greatly influenced by the direction and duration of cell enlargement (Braidwood et al., 2014). Plant cells predominantly show anisotropic expansion, where the rate and direction of growth vary across the cell surface, allowing cells to acquire a variety of forms to limit the magnitude of mechanical stress and simultaneously enhance tissue strength (Johnson and Lenhard, 2011; Braidwood et al., 2014; Sapala et al., 2018). Anisotropic cell growth can be either diffused type where expansion is dispersed over the cell surface, or tip-growth type where expansion occurs only at localized tips (Martin et al., 2001). For example, cells in hypocotyl, stem, and root show growth along the longitudinal direction at their side walls over the entire surface. By contrast, root hairs and pollen tubes exhibit restricted growth only at a single site at the cell tip (Braidwood et al., 2014). In yet another growth pattern, the pavement cells in leaves and cotyledons show multiple growth polarities on segments of their surface, ultimately producing puzzle piece-shaped cells with lobes and indentations. We refer to this type of cell growth as planar growth since the expansion is primarily in the *X*–*Y* plane, with little expansion in the orthogonal direction. Distinct cytological properties such as differential deposition of cell-wall material, orientation of cellulose microfibrils, and polarized accumulation of cytoplasmic content determine the mode of cell growth and organ morphology (Braidwood et al., 2014). However, certain cells such as parenchyma of potato tuber or apple fruit display a diffused isotropic mode of growth, forming isodiametric cells compacted together where the cellulose microfilaments are randomly oriented (Cosgrove, 2014).

In most tissue types, rapid increase in cell volume is directly associated with endoreplication cycle where DNA is replicated without cytokinesis or chromosome segregation, leading to enhanced nuclear ploidy level (Melaragno et al., 1993; Edgar and Orr-Weaver, 2001; Breuer et al., 2010). Polyploidization is known to promote ribosome biogenesis, resulting in an increase in protein synthesis and in total cytoplasmic content, which ultimately leads to cell growth (Larkins et al., 2001; Sugimoto-Shirasu and Roberts, 2003). Thus, modulating the onset of endocycle and the duration of its progression substantially influence organ size with a strong correlation with cellular ploidy level. FASCIATA1 (FAS1), a chromatin assembly subunit protein, and E2F TARGET GENE 1 (ETG1), a replisome factor, interfere in the chromatin assembly that triggers endoreplication in plants. Consequently, *fas1* and *etg1* mutants perform additional rounds of endocycle, leading to larger cells and organs (Ramirez-Parra and Gutierrez, 2007; Takahashi et al., 2010). Similarly, cell cycle inhibitory *KIP-RELATED PROTEIN* (*KRP*) genes controlling endoreplication and chromatin structure regulate expression of genes required for cell expansion (Jegu et al., 2013). The quintuple *krp* mutant exhibits leaves with smaller epidermal cells and *KRP*-overexpression lines form larger cells, suggesting a direct correlation of KRP abundance and endoreplication-mediated cell growth (Verkest et al., 2005; Cheng et al., 2013). Additional rounds of endoreplication in dark-grown hypocotyl cells serve as a major determinant of de-etiolated seedling growth in contrast to light-grown seedlings (Gendreau et al., 1997). In floral organs, suppressing the transition to endoreduplication in the *frill1* mutant, which has a reduced sterol methyltransferase activity, results in expanded cells with enlarged nuclei during the late stage of petal development (Hase et al., 2000). It is also suggested that the combination of phytohormones, nutrients, and environmental signals trigger endoreplication and cell growth (Kondorosi et al., 2000; Sugimoto-Shirasu and Roberts, 2003). For instance, gibberellic acid (GA) and ethylene coordinate with DNA synthesis and endoreplication-mediated hypocotyl cell elongation (Gendreau et al., 1999). Many reports in multiple plant species strongly indicate the presence of endocycle-independent mechanism of cell growth affecting organ size (Sugimoto-Shirasu and Roberts, 2003).

## Mechanism of Cell Growth

The rigid wall of plant cells – comprised of complex meshwork of cellulose, hemicellulose, pectin, and glycoproteins – accommodates two seemingly contradictory biological functions - providing a strong structural support to the cell and an adjustable elasticity necessary for its growth (Bashline et al., 2014). The turgor pressure triggered by osmotic activity leads to cell-wall loosening and produces enlarged cells with balanced water potential without losing their integrity (Cosgrove, 2005; Seifert and Blaukopf, 2010; Bashline et al., 2014). The physical properties of cell-wall are altered to promote expansion by the activity of several cell-wall-modifying enzymes such as glycosyltransferases, methylesterases, hydroxylases, and hydrolases. These enzymes target major components of the cell wall (Braidwood et al., 2014). For example, the EXPANSIN (EXP) family proteins promote cell-wall loosening by breaking the non-covalent bonds between cellulose microfibrils in an acidic environment and correspondingly produce bigger leaves with larger cells when overexpressed (Cho and Cosgrove, 2000; Marowa et al., 2016). Similarly, pectin methyl esterases hydrolyze ester bond and enhance negative electrostatic charge within the cell wall to provide flexibility and mobility to homo-galacturonan in the cell wall. Mutant analysis of multiple *cellulose synthase* (*CESA*) genes has demonstrated the redundant function of these cell-wall synthesis promoting genes in cell expansion in multiple organs (Burn et al., 2002).

Phytohormones play a critical role in promoting cell growth in association with either cell-wall-remodeling enzymes or by enhancing orientated deposition of cellular microfibrils (Wolters and Jurgens, 2009). In addition, auxin, brassinosteroid (BR), and GA promote cell-wall biosynthesis and expansion by activating several regulatory transcription factors. Auxin induces rapid cell expansion in stem, hypocotyl, leaf, and coleoptile by activating a proton (H^+^) pump ATPase in the plasma membrane (PM), resulting in extracellular acidification, an environment favoring the cell-wall loosening enzymes (Rayle, 1973; Rayle and Cleland, 1977, 1992; Perrot-Rechenmann, 2010). The early auxin-responsive *SMALL AUXIN UPREGULATED RNA* (*SAUR*) gene family members participate in modulating PM H^+^-ATPase activity through direct interaction and inhibition of PP2C-D protein phosphatases (Spartz et al., 2014). Recent reports have shed light on tissue-specific expression pattern of *SAUR* genes and their involvement in distinct morphological processes under the regulation of developmental and environmental factors (Ren and Gray, 2015; Stortenbeker and Bemer, 2019). BR, GA, jasmonic acid (JA), and abscisic acid (ABA) also regulate SAUR-mediated cell growth (Ren and Gray, 2015). Auxin, BR, and GA integrate environmental signals through the DELLA-ARF6-BZR1-PIF4 complex to form a central regulatory network and maintain cell elongation by regulating an overlapping set of *SAUR* genes (Oh et al., 2014; Van Mourik et al., 2017). Mutants deficient in *AUXIN RESPONSE FACTORS* (*ARF*s) form shorter hypocotyl compared to the wild type, further supporting the role of auxin response in cell expansion (Reed et al., 2018). Another homeodomain transcription factor HAT2 is also known to promote cell elongation in hypocotyl in response to auxin signaling (Sawa et al., 2002). A recent report suggests that increased endogenous auxin, triggered by a low-sugar state in the cotyledons following seed germination, promotes compensation-mediated cell enlargement (Tabeta et al., 2021). Another recent study demonstrates that constitutively activated salicylic acid (SA) signaling suppresses compensation-induced cell expansion in leaves, thus assigning a role for SA in organ size regulation (Fujikura et al., 2020).

The regulators of the cell-wall extensibility factors described above are studied in less detail. The *ARGOS-LIKE* (*ARL*) gene is preferentially expressed in cotyledon, mature root, and leaves and promotes organ size as a result of increased cell expansion (Hu et al., 2006). Similarly, overexpression of ZINC-FINGER HOMEODOMAIN 5 (ZHD5) produces bigger leaves with enlarged epidermal cells by acting on yet unidentified target genes required for cell expansion (Hong et al., 2011). Few more examples of cell expansion regulators include TARGET OF RAPAMYCIN (TOR), CELL CYCLE SWITCH PROTEIN 52 (CCS27A), CYTOCHROME P450, FAMILY 78, SUBFAMILY A (CYP78A6), and ERBB-3 BINDING PROTEIN 1 (EBP1), overexpression of which results in larger organs due to increased cell expansion (Gonzalez et al., 2012; Vanhaeren et al., 2015). The list of the transcription factors that directly or indirectly regulate cell-wall expansion is still growing, with the recent addition of the TEOSINTE BRANCHED1, CYCLOIDEA, PROLIFERATING CELL FACTOR (TCP) family proteins.

## The Teosinte Branched1, Cycloidea, Proliferating Cell Factor Transcription Factors in Post-Mitotic Cell Growth

Teosinte branched1, cycloidea, proliferating cell factor proteins participate in sequence-specific DNA-binding, transcriptional activation, and protein–protein interaction to regulate diverse developmental processes including flower symmetry, leaf and petal morphogenesis, trichome development, axillary branching, and pathogen defense (Martin-Trillo and Cubas, 2010; Sugio et al., 2014; Danisman, 2016; Sarvepalli and Nath, 2018; Challa et al., 2019). They are distinguished by their non-canonical basic helix-loop-helix (bHLH) domain known as the TCP domain and are present from chlorophyte algae to higher land plants (Navaud et al., 2007; Dhaka et al., 2017). Based on sequence diversity in amino acid residues within the TCP-domain, the 24 TCP proteins encoded by the Arabidopsis genome are divided into the class I subfamily with thirteen members and the class II subfamily with eleven members (Kosugi and Ohashi, 2002; Li, 2015). Eight class II TCPs are collectively called CINCINNATA-like TCPs (CIN-TCPs) that form a subclade and are expressed in the transition zone in leaf primordia where they redundantly suppress proliferation and promote differentiation in dividing cells (Nath et al., 2003; Das Gupta et al., 2014; Sarvepalli and Nath, 2018; Challa et al., 2019). Transcripts of five *CIN-TCP* genes – *TCP2, 3, 4, 10*, and *24* – are degraded by the microRNA miR319 (Palatnik et al., 2003), adding an additional level of complexity in CIN-TCP-mediated regulation of leaf morphogenesis. In addition to leaf maturation, CIN-TCPs also regulate leaflet initiation, biotic and abiotic stress tolerance, photomorphogenic seedling growth, phytohormone signaling, flowering time control, cell-wall thickening, etc (Koyama et al., 2007, 2010a, 2017; Navaud et al., 2007; Efroni et al., 2008; Schommer et al., 2008; Sarvepalli and Nath, 2011a; Aguilar-Martinez and Sinha, 2013; Das Gupta et al., 2014; Wang et al., 2014, 2015; Challa et al., 2016, 2019; Kubota et al., 2017; Lei et al., 2017; Zhou et al., 2018, 2019; Fan et al., 2020; Fang et al., 2021). Most developmental processes regulated by CIN-TCPs are linked to their involvement in suppressing proliferation and promoting differentiation of cells. Effects of CIN-TCPs on the exit from division cycle within leaf primordia have been reviewed elsewhere (Sarvepalli and Nath, 2018).

Accumulating evidence in recent years has revealed a direct participation of CIN-TCPs in post-mitotic cell expansion, which could not be elucidated earlier due to extensive functional redundancy among the members, making the loss-of-function analysis less effective (Efroni et al., 2010; Sarvepalli and Nath, 2011a; Koyama et al., 2017). On the other hand, overexpression of miR319-resistant CIN-TCPs in their endogenous expression domain causes embryonic and seedling lethality, making gain-of-function analysis challenging (Palatnik et al., 2003; Koyama et al., 2007). Moreover, a change in cell proliferation in leaf primordia is often accompanied by an opposite change in cell size by a phenomenon called compensation, making the analysis of gene function in cellular regulation difficult to interpret (Horiguchi and Tsukaya, 2011; Hisanaga et al., 2015). Considering the contribution of CIN-TCPs in repressing cell division potential (Challa et al., 2019), it is rather a daunting task to uncouple TCP-dependent cell expansion from compensatory cellular growth in post-embryonic organs such as leaves.

Several class I TCP proteins have also been implicated in cell division and cell growth. For example, TCP20 is enriched in the chromatin regions that encode cyclin CYCB1;1 and several ribosomal proteins, suggesting its role in coupling division and growth of cells (Li et al., 2005). However, we focus on the class II TCP proteins in this review and set aside the discussion on the class I proteins for future.

## CINCINNATA-Like TCP-Mediated Cell Growth in Embryonic Organs

### Cell Elongation in Hypocotyl

Hypocotyl initials are formed by patterned cell division during embryo development. Upon seed germination and seedling establishment, hypocotyl grows in length solely by directional cell expansion (Nemhauser and Chory, 2002; Boron and Vissenberg, 2014). Several studies on hypocotyl growth at the cellular level suggest an extreme elongation of the under-differentiated epidermal cells (up to 100-fold elongation compared to embryonic cell stage) as a result of rounds of endoduplication under darkness as opposed to the light-grown seedlings (Gendreau et al., 1997). The epidermal cells in hypocotyl follow a steep acropetal mode of growth where elongation starts at the basal cells and gradually moves upward (Miséra et al., 1994; Wei et al., 1994; Gendreau et al., 1997).

**FIGURE 1:**
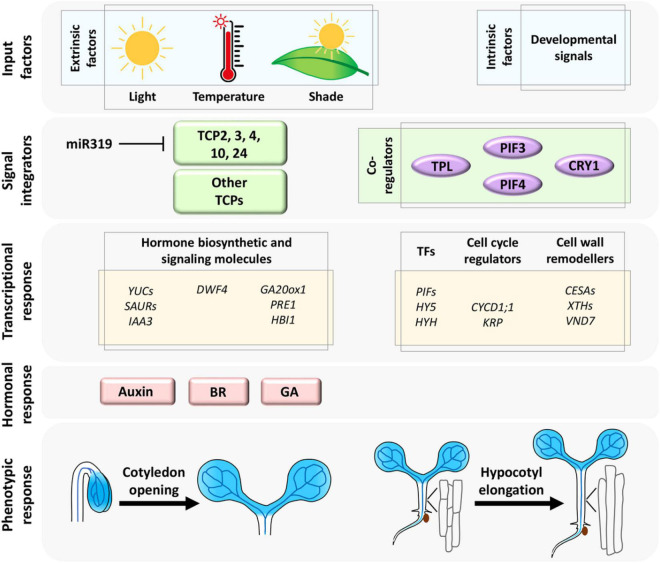
The extrinsic (environmental) and intrinsic (developmental) signals are consolidated by miR319-regulated CIN-TCP transcription factors and their co-regulators (Dong et al., 2019; Ferrero et al., 2019; Zhang et al., 2019; Zhou et al., 2019) to form a regulatory network that activates downstream target molecules and phytohormones to promote cell growth linked to hypocotyl elongation and cotyledon opening. Shaded blue color represents *CIN-TCP* gene expression pattern.

Both miR319-targeted and non-targeted CIN-TCP proteins promote hypocotyl elongation under the influence of environmental signals (Figure 1). Analysis of loss and gain-of-function mutants has demonstrated that miR319-regulated CIN-TCPs promote hypocotyl elongation by directly activating *YUCCA5* (*YUC5*)-mediated auxin biosynthesis, which in turn activates the central ARF6/8-BZR1 circuit and downstream cell expansion genes (Challa et al., 2016). TCP1, a non-CIN-TCP class-II TCP protein, also enhances cell elongation in hypocotyl by directly activating the BR biosynthetic gene *DWARF4* (Guo et al., 2010), suggesting that the class II TCPs promote hypocotyl cell growth by acting on multiple phytohormone pathways. Interestingly, TCP3, another miR319-regulated CIN-TCP member, is reported to activate *INDOLE-3-ACETIC ACID3/SHORT HYPOCOTYL2 (IAA3/SHY2)*, a negative regulator of auxin signaling, though overexpression of a miR319-resistant form of TCP3 causes elongation of hypocotyl cells (Koyama et al., 2010a). These observations imply distinct functions of redundant CIN-TCP partners in promoting cell growth, perhaps to maintain homeostasis in hormone response.

Though CIN-TCPs promote hypocotyl elongation, their promoters are primarily active in the cotyledons (Koyama et al., 2007; Challa et al., 2016; Dong et al., 2019). It is possible that these proteins exert a non-cell-autonomous effect on hypocotyl elongation through systemic enhancement of cotyledon-derived auxin response. This is supported by the fact that polar auxin transport is required to promote hypocotyl elongation in light-grown seedlings, though not in dark-grown seedlings (Jensen et al., 1998). Much of the environmental cues are sensed by the cotyledons where most auxin biosynthesis, response, and transport genes are expressed, and this cotyledon-derived mobile auxin travels to hypocotyl where it promotes cell elongation, as identified by chemical intervention and surgical experiments (Tao et al., 2008; Keuskamp et al., 2010; Procko et al., 2014). Effects of mobile auxin is seen primarily in the epidermal tissue where auxin interacts with the BR pathway to induce hypocotyl growth (Procko et al., 2016). In addition to CIN-TCPs, several class-I TCP members also promote hypocotyl elongation (listed in Table 1) in a context and condition-dependent manner.

**TABLE 1:** *TCP* genes implicated in cell growth in *Arabidopsis thaliana* embryonic organs.

*TCP* genes	*TCP* class	Organ	Function	References
*TCP20*	I	Hypocotyl Root	Reduces cell elongation by targeting cellulose synthase genes	Herve et al., 2009
*TCP1*	I	Hypocotyl Petiole	Promotes hypocotyl elongation by activating BR-biosynthetic gene *DWARF4*	Guo et al., 2010
*TCP14, 15*	I	Hypocotyl	Induce GA-biosynthetic gene *GA20ox1* and cell expansion regulators *PRE1*, *HBI1*	Ferrero et al., 2019
*TCP7, 8, 14, 15, 21, 22, 23*	I	Hypocotyl	Promote endoreplication-mediated cell expansion by directly activating *CYCD1;1*	Zhang et al., 2019
*TCP14, 15*	I	Hypocotyl	Enhance cell elongation by direct activation of several auxin-response genes	Ferrero et al., 2021
*TCP3*	II	Hypocotyl	Elongates hypocotyl when miR319-resistant version is overexpressed	Koyama et al., 2007
*TCP3*	II	Hypocotyl Cotyledon	Suppresses auxin response by activating *IAA3/SHY2* and *SAUR* genes	Koyama et al., 2010a
*TCP4*	II	Hypocotyl Cotyledon	Promotes hypocotyl elongation when hyperactivated	Sarvepalli and Nath, 2011a
*TCP24*	II	Hypocotyl	Represses photomorphogenic growth in miR319-dependent manner	Tsai et al., 2014
*TCP2*	II	Hypocotyl Cotyledon	Promotes photomorphogenic growth by activating *HY5* and *HYH*	He et al., 2016, 2021
*TCP4, 2, 3, 10, 24*	II	Hypocotyl	Enhance hypocotyl cell elongation by directly activating *YUC5*	Challa et al., 2016
*TCP17*	II	Hypocotyl	Promotes shade-induced hypocotyl growth by activating *PIF*s and *YUC*s	Zhou et al., 2018
*TCP17, 5, 13*	II	Hypocotyl	Promote thermoresponsive hypocotyl growth by physical interaction with PIF4 and CRY1	Zhou et al., 2019
*TCP5, 13, 17*	II	Hypocotyl	Promote thermomorphogenic hypocotyl elongation along with PIF4	Han et al., 2019
*TCP4*	II	Cotyledon	Promotes cotyledon opening through *SAUR* genes during seedling de-etiolation	Dong et al., 2019

### Cell Expansion in Cotyledons

Planar expansion of pavement cells with interdigitation has been widely studied using cotyledon as a model organ due to their simplicity and lack of concomitant cell division. Hyperactivated TCP4 promotes cotyledon cell expansion (Sarvepalli and Nath, 2011a). The organ-specific activity of the early auxin response genes *SAUR16* and *SAUR50* contribute to differential cellular response in cotyledon opening, which is driven by cell expansion and hypocotyl elongation upon enhancement in TCP4 level during seedling de-etiolation (Dong et al., 2019). *SAUR16* and *SAUR50* act as potential direct targets of TCP4 and this transcriptional activity of TCP4 is inhibited by PHYTOCHROME INTERACTING FACTOR3 (PIF3), a canonical bHLH transcription factor abundantly expressed in etiolated seedlings (Dong et al., 2019). PIF3 is recruited to the *SAUR* promoter and interferes with DNA-binding by TCP4 to retain closed unexpanded cotyledons under darkness. TCP4 does not seem to physically interact with PIF3 in this process, and thus the exact mechanism of this competitive binding to *SAUR* promoter is still an open question. By acting on the ARF-BZR1 signaling node and *SAUR* genes, CIN-TCPs promote cotyledon cell expansion perhaps by cell-wall remodeling, though evidence in support of this proposition has remained elusive. Distinct phytohormones such as GA, ethylene, and ABA also regulate cotyledon cell expansion independently (Collett et al., 2000; Stoynova-Bakalova et al., 2004). Crosstalk between CIN-TCPs and these hormone response pathways are not well studied. Based on the involvement of CIN-TCPs in transcriptional regulation of multiple phytohormones (Guo et al., 2010; Sarvepalli and Nath, 2011b; Challa et al., 2016; Gonzalez-Grandio et al., 2017), one can speculate their participation as signal integrators, which can be further explored (Figure 1).

## CINCINNATA-Like TCP-Mediated Cell Growth in Post-Embryonic Organs

Like cotyledons, post-embryonic lateral organs such as leaves also display planar growth driven by cell expansion in both transverse and longitudinal axes. Phytohormones such as ethylene and cytokinin promote cell growth along the transverse axes, whereas auxin, BR, and GA promote expansion along longitudinal axes (Mizukami, 2001). These hormones, individually or in combination, affect the organization of cellular and cortical cytoskeleton elements leading to differential expansion rate along growth axes (Blume et al., 2012). In addition, hormones also impact tubulin and actin transcript abundance (Kandasamy et al., 2001; Blume et al., 2012).

### Cell Growth in Leaf

**FIGURE 2:**
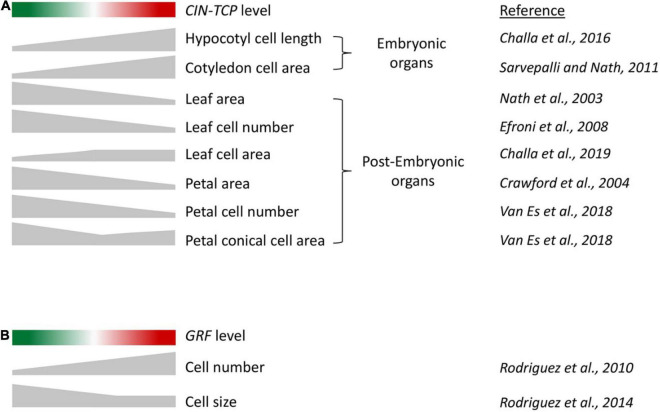
**(A)** A schematic to describe the correlation between CIN-TCP activity and cell growth in embryonic and post-embryonic organs. Red and green shades indicate progressively higher and lower *CIN-TCP* levels, respectively, compared to wild-type level in the middle. Height of the gray-shaded objects corresponds to the extent of phenotype indicated on the right. Organs with embryonic origin such as cotyledons and hypocotyl where cell division and expansion are temporally exclusive, exhibit cell expansion directly proportional to *CIN-TCP* level. In post-embryonic lateral organs such as leaves and petals where cell division and expansion are contemporary, the correlation of cell growth and *CIN-TCP* abundance is more complex due to combined action of proliferation and expansion. **(B)** A schematic describing the correlation of GRF activity and cell number and size in leaf.

As mentioned earlier, analysis of gene function on cell growth in leaf primordium is compounded by compensation wherein an alteration in cell number is compensated by an opposite effect on cell area, resulting in a tendency of the final leaf size to remain unaffected. Genetic analysis coupled with growth kinematic studies have revealed the effect of CIN-TCP proteins on cell growth (Challa et al., 2019). Loss-of-function mutants of CIN-TCPs form bigger leaves with excess cells, and their overexpression results in smaller leaves with fewer cells (Figure 2A; Efroni et al., 2008; Schommer et al., 2014; Challa et al., 2019). Increased cell number in *cin-tcp* loss-of-function mutants is accompanied by smaller cells, suggesting a compensatory effect. Though the cell-size defect is corrected to the wild-type level when a dominant form of TCP4 is activated, the cell size does not increase beyond the wild-type level, indicating that CIN-TCPs are required but not sufficient for pavement cell growth. These cell kinematics results have been interpreted as CIN-TCPs promoting the division-to-differentiation switch in the proliferating leaf pavement cells (Challa et al., 2019). According to this interpretation, reduced CIN-TCP function causes fewer cells to depart the division cycle and enter the expansion phase, resulting in excess cells at maturity with a higher proportion of smaller cells. On the contrary, gain of CIN-TCP function causes more cells to depart cell cycle and enter differentiation, leaving fewer cells to divide. This interpretation appears to explain both the loss and gain-of-function phenotypes of *CIN-TCP* mutants without ascribing a cell growth function to them. It also partly explains the cellular basis of compensation in mature leaves: prolonging the proliferation phase accumulates more cells of smaller size, measuring the average cell area at maturity smaller than that in wild type. CIN-TCP proteins possibly promote the division-to-differentiation transition by increasing the nuclear ploidy level via endoreplication (Challa et al., 2019; Zhang et al., 2019). Transition to cell differentiation by CIN-TCPs is redundant with the NGATHA and class II KNOTTED1-LIKE transcription factors, and higher order mutants of these genes produce persistent leaf growth with delayed differentiation status (Alvarez et al., 2016; Challa et al., 2021). The inability of CIN-TCPs to increase cell size over and above the wildtype level can also be interpreted as a threshold-dependent activity below which they are unable to promote cell expansion. Alternatively, it is also possible that CIN-TCPs depend on other factors for driving cell expansion in leaf. Cell-size analysis using transgenic lines expressing a higher level of *CIN-TCP* would test these possibilities.

The miR396-*GRF* module regulates leaf size and influences cell proliferation in a manner opposite to that of miR319-CIN-TCP. The GRF transcription factors, which are repressed by miR396, promote cell proliferation in the leaf primordia (Rodriguez et al., 2010; Schommer et al., 2014). Leaves overexpressing GRF accumulate excess pavement cells, whereas GRF down-regulation leads to fewer cells (Rodriguez et al., 2010; Challa et al., 2019). These results are in agreement with a molecular link between these two modules where the miR319-*CIN-TCP* module acts as an upstream regulator of miR396-*GRF*, with CIN-TCPs directly activating *MIR396* transcription (Schommer et al., 2014). However, contrary to the effects of CIN-TCPs on cell size, loss of *GRF*s leads to bigger pavement cells whereas their gain-of-function fails to alter cell size (Kim and Lee, 2006; Rodriguez et al., 2010, 2014; Horiguchi and Tsukaya, 2011). These results suggest a diverging effect of CIN-TCPs and GRFs on cell size regulation (Figure 2B).

At the molecular level, only a few cell-expansion genes have been identified as targets of TCP proteins, which control cell growth in lateral organs (listed in Table 2). For instance, TCP4 induces pavement cell differentiation by elevating auxin response as well as by directly activating the HOMEODOMAIN *ARABIDOPSIS THALIANA* 1 (HAT2)-encoding gene in an auxin-independent manner (Challa et al., 2019). A recent report suggests that the miR319-non-targeted *CIN-TCP*s such as *TCP5*, *13*, and *17* reduces cell expansion; TCP13 directly associates with the upstream regulatory region of *Arabidopsis thaliana homeobox 12* (*ATHB12*), a known cell expansion gene, and reduces its transcription (Hur et al., 2019). Mutation in the class-I *TCP* member *TCP20* results in enlarged pavement cell during early leaf development, which is antagonistic to the effect shown by their class-II counterparts (Danisman et al., 2012).

**TABLE 2:** *TCP* genes associated with post-mitotic cell growth in lateral organs.

Genes	Class	Organ	Plant species	Function	References
*TCP1*	I	Leaf Stem Petiole	*Arabidopsis thaliana*	Promotes cell elongation	Koyama et al., 2010b
*TCP20*	I	Leaf	*Arabidopsis thaliana*	Inhibits pavement cell size without affecting leaf size	Danisman et al., 2012
*GhTCP14*	I	Cotton fiber	*Gossypium hirsutum*	Promotes auxin-dependent elongation of cotton fiber cells	Wang et al., 2013
*TCP7, 8, 22, 23*	I	Leaf Petiole	*Arabidopsis thaliana*	Promote cell growth and suppress proliferation	Aguilar-Martinez and Sinha, 2013
*CmTCP14*	I	Petal Leaf	*Chrysanthemum morifolium*	Inhibits cell area and organ size by interacting with DELLA proteins	Zhang et al., 2017
*TCP7, 8, 14, 15, 21, 22, 23*	I	Leaf	*Arabidopsis thaliana*	Promote endoreduplication-dependent cell expansion in leaf	Zhang et al., 2019
*TCP15*	I	Stamen filament	*Arabidopsis thaliana*	Promotes cell elongation through *SAUR63* subfamily genes	Gastaldi et al., 2020
*CsBRC1-like*	I	Leaf	*Cucumis sativus* L.	Suppresses cell differentiation through auxin and cytokinin signaling	Shen et al., 2021
*CIN*	II	Petal Leaf	*Antirrhinum majus*	Controls cell differentiation and growth in leaf and petal	Crawford et al., 2004
*CIN-TCPs*	II	Leaf	*Arabidopsis thaliana*	Promote cell growth and leaf maturation	Efroni et al., 2008
*CIN-TCPs*	II	Leaf	*Arabidopsis thaliana*	Promote cell differentiation through auxin, *miR164*, *SHY2* and *SAURs*	Koyama et al., 2010a
*TCP4*	II	Leaf	*Arabidopsis thaliana*	Promotes onset of differentiation with larger cells when hyperactivated	Sarvepalli and Nath, 2011a
*CIN*	II	Leaf	*Antirrhinum majus*	Accelerates cell maturity through *HK4* and *IAA3*/*SHY2*-like genes	Das Gupta et al., 2014
*TCP5*	II	Petal	*Arabidopsis thaliana*	Suppresses conical cell growth by inhibiting ethylene signaling	Van Es et al., 2018
*TCP13*	II	Leaf	*Arabidopsis thaliana*	Suppresses leaf cell growth by repressing *ATHB12*	Hur et al., 2019
*TCP4, 2, 3, 10, 24*	II	Leaf	*Arabidopsis thaliana*	Promote commitment to differentiation in mitotic cells	Challa et al., 2019

Loss of miR319-targeted *CIN-TCP*s in the *MIR319A*-overexpressing leaves, in addition to causing smaller cell size, also reduces the typical jigsaw-puzzle morphology, making the pavement cells relatively rounder (Efroni et al., 2008). The fact that CIN-TCPs transcriptionally regulate several phytohormones which are necessary for intercalated expansion of pavement cells might provide a mechanistic insight to this observation. Small non-lobed pavement cells also appear in the mutant of the chromatin remodeler SWI/SNF ATPase protein BRAHMA, which is a well-established protein interactor of TCP4, indicating an epigenetic regulation of cell expansion (Efroni et al., 2013; Vercruyssen et al., 2014; Thouly et al., 2020).

### Cell Growth in Petal

The petals of the *cin* mutant in snapdragon are made of fewer epidermal cells that are larger in size compared to wild type, suggesting that *CIN-TCPs* exert an opposite effect on cell expansion in petals to their effect on leaf (Crawford et al., 2004). TCP5, along with TCP13 and TCP17, also suppresses the area of the conical cells in Arabidopsis petals by inhibiting ethylene biosynthesis through direct binding to the *1-aminocyclopropane-1-carboxylate* (*ACC*) *synthase 2* (*ACS2*) locus (Van Es et al., 2018). The *tcp5; tcp13; tcp17* triple mutant forms bigger and broader petals with excess and larger conical cells, suggesting an inhibitory effect of these genes on both cell division and expansion, as opposed to the compensatory effect observed in leaves. Conversely, *TCP5-*overexpressing lines form smaller petals with fewer cells (Van Es et al., 2018). However, the conical cells in these *TCP5* gain-of-function petals are slightly larger than wild type, perhaps due to compensation.

### CINCINNATA-Like TCPs Regulate Cell Wall Composition

CINCINNATA-like TCP members such as TCP4 are recruited to the genomic regions of several genes involved in cell-wall biogenesis and lignin deposition and promote secondary cell-wall thickening in diverse species. For instance, activation of miR319-resistant TCP4 triggers transcription of a xylem differentiation-promoting gene *VASCULAR-RELATED NAC-DOMAIN7* (*VND7*), and several cellulose (*CESA4*, *7*, and *8*) and lignin (*LAC4* and *LAC17*) biosynthetic genes (Sun et al., 2017). Overexpression of GhTCP4 promotes cell-wall thickening by binding to the *cis* elements of secondary cell-wall biosynthetic genes such as *GhCESA7* and *GhFSN1* in cotton (Cao et al., 2020). On the contrary, TCP24 negatively regulates genes involved in secondary cell-wall biogenesis and thickening, indicating a possible involvement in cell-wall remodeling and extensibility (Wang et al., 2015). In summary, these results point toward a new field of study which can connect CIN-TCPs directly to the cell-wall biochemistry and provide a mechanistic basis of TCP-mediated cell expansion.

## CINCINNATA-Like TCPs Translate Environmental Signal into Cell Growth Response

Recent findings implicate a role for the *CIN-TCP* genes in modulating hypocotyl elongation in response to environmental cues such as light and temperature. Multiple alleles of the *jaw-D* dominant mutation, where overexpression of miR319 downregulates five cognate *CIN-TCP* targets (*TCP2, 3, 4, 10*, and *24*), show light-hypersensitive effect, where TCP4 and TCP24 suppress photomorphogenesis and promote hypocotyl elongation (Tsai et al., 2014). By contrast to TCP4 and TCP24, TCP2 inhibits hypocotyl elongation specifically under blue light condition by interacting with the blue-light receptor CRYPTOCHROME 1 and activating the transcription of *ELONGATED HYPOCOTYL5* (*HY5*) and *HY5-HOMOLOG*, genes that promote photomorphogenesis (He et al., 2016). The non-miR319-targeted CIN-TCP members TCP5, TCP13, and TCP17 also promote hypocotyl elongation in response to canopy-shade by directly activating the auxin biosynthetic *YUC* genes and *PIF4/5* (Zhou et al., 2018). *TCP5/13/17* promote seedling growth also in response to high temperature by targeting PIF4 activity at both transcriptional and post-transcriptional level (Han et al., 2019; Zhou et al., 2019). Interestingly, *TCP5* is expressed in both cotyledon and hypocotyl where it directly enhances local increase in auxin biosynthesis and BR response upon elevated temperature (Bellstaedt et al., 2019; Han et al., 2019). Heat treatment shifts *TCP5* expression from leaf blade to petiole, supporting its role in differential cell expansion of petiole and reduced leaf size (Han et al., 2019). Thus, there appears to be a positive correlation between the level of CIN-TCP expression and the extent of cell elongation in embryonic organs such as hypocotyl (Figure 2A). Environmental challenges such as low light intensity and rise in ambient temperature induce unequal growth rates between adaxial and abaxial regions of the petiole cells, leading to hyponastic growth in association with major phytohormones (Millenaar et al., 2009). Chimeric repression of the CIN-TCP member TCP3 shows irregularly differentiated petiole formation, whereas overexpression of *TCP5/13/17* displays longer petiole in response to high temperature, consistent with their petiole-specific expression (Koyama et al., 2007; Han et al., 2019). Further, enhanced and reduced activity of TCP1 leads to elongated and shortened petiole, respectively, which is mediated by auxin and BR response (Guo et al., 2010; Koyama et al., 2010b).

## Conclusion and Outlook

The apparent divergent effects of CIN-TCP transcription factors on the morphogenesis of various primordia including flower, axillary meristem, leaf, and petal converge at their inhibitory effect on cell proliferation. This effect is at least in part mediated by directly activating the transcription of *KRP* cell-cycle inhibitor genes. By advancing the cessation of cell proliferation and consequently triggering differentiation in various primordia, these proteins regulate the shape and size that are manifested in the mature organs. This rather simplified effect of these proteins has now started to expand with the identification of several target genes, interacting partners, and upstream regulatory proteins, presenting a complex regulatory network involving these transcription factors. One such effect is on cell growth of embryonic organs, which grow solely by cell expansion post-germination. Elongation of hypocotyl cells by CIN-TCPs can be explained by YUCCA-mediated auxin biosynthesis at the shoot apex that is transported to the growing hypocotyl (Challa et al., 2016). Though activation of dominant CIN-TCPs within the cell proliferation phase of leaf primordia reduces cell number, their activation beyond the proliferation phase has no effect on the cell or leaf size (Challa et al., 2019). This is perhaps not surprising, considering that the CIN-TCP genes are primarily expressed in developing leaves. However, the failure of the CIN-TCPs to increase cell expansion in leaves is rather intriguing.

Studies emerging over the past decade have elucidated the function of the CIN-TCP proteins as central integrators of environmental, developmental, and hormonal signals to perform several key developmental processes. By participating at multiple stages of organ growth including the duration and extent of cell proliferation, cell-fate transition from division to differentiation, onset of post-mitotic cell growth and scope of cell expansion, CIN-TCPs program diverse organs to achieve final size and shape over a wide range of plant species. To overcome the intertwined relationship between cell division and differentiation, and to study the exact contribution of *CIN-TCP* genes in cell expansion *per se*, the embryonic organs such as hypocotyl and cotyledons are useful model organs. However, the sequence of molecular events leading to cell enlargement and the exact kinetics of cell elongation in association with *TCP* genes awaits detailed elucidation. Finally, understanding the conservation of this CIN-TCP-dependent modulation in post-mitotic cellular growth across species is a challenge for future studies, considering organ size is a major contributor to crop productivity.

## Author Contributions

MR wrote the first draft of the manuscript and progressively modified it to the final version. KC and KS contributed to writing and corrections. UN corrected the manuscript and guided the other authors to produce the final version. All authors contributed to the article and approved the submitted version.

## Conflict of Interest

The authors declare that the research was conducted in the absence of any commercial or financial relationships that could be construed as a potential conflict of interest.

## Publisher’s Note

All claims expressed in this article are solely those of the authors and do not necessarily represent those of their affiliated organizations, or those of the publisher, the editors and the reviewers. Any product that may be evaluated in this article, or claim that may be made by its manufacturer, is not guaranteed or endorsed by the publisher.
